# Novel definition of textbook outcome in biliary system cancers and its influence on patients' survival and quality of life

**DOI:** 10.1002/cam4.7186

**Published:** 2024-04-10

**Authors:** Manar Mikhail Atyah, Li Xu, Zhiying Yang

**Affiliations:** ^1^ Department of Hepatobiliary and Pancreatic Surgery China–Japan Friendship Hospital Beijing China

**Keywords:** biliary system cancer, quality of life, surgery, survival, textbook outcome

## Abstract

**Background:**

The definition of textbook outcome in biliary system cancers is a developing concept in need of expansion and investigation of its association with survival and quality of life.

**Methods:**

In this original research, we developed a novel “all or none” textbook outcome definition which addresses the rapid recovery of post‐surgical indexes, in addition to short‐term mortality, hospital re‐admission, prolonged stay, surgical margin and postoperative complications. Based on the fulfillment of relevant criteria, patients were divided into textbook outcome and non‐textbook outcome groups and their characteristics and survival data were analyzed. A customized “quality of life” questionnaire was developed to address short‐term recovery and post‐discharge life quality of patients. Association with quality of life improvement was then investigated.

**Results:**

A total of 129 patients were included. Textbook outcome was achieved in 25.58% of patients (37.04% of gallbladder cancer patients and 17.8% of cholangiocarcinoma patients). Compared to non‐textbook outcome group, patients with textbook outcome had lower rate of pre‐operative biliary drainage (*p* = 0.026), higher rate of normal preoperative liver function (*p* < 0.001) and tumor markers (*p* = 0.001), reduced perioperative bleeding (*p* = 0.006) and blood transfusion (*p* = 0.005), and higher rate of N0 stage cases (*p* = 0.008). Textbook outcome was also associated with enhanced survival, significantly in older patients (<65 years) (1‐year survival rate: 100% vs. 78.57% (*p* = 0.108), 2‐year survival rate: 87.5% vs. 44% (*p* = 0.046)). Finally, textbook outcome was significantly associated with enhanced basic daily performance (*p* < 0.001), social life performance (*p* = 0.033), and personal evaluation (*p* < 0.001), and thus improved quality of life (*p* < 0.001).

**Conclusions:**

The novel definition of textbook outcome was able to address the specific nature of recovery after resection of biliary system cancers. Expanding the scope of textbook outcome and addressing the influence on survival and quality of life provides a comprehensive concept able to reflect physical, psychological and functioning enhancements in patients recovery.

## 1 INTRODUCTION

The biliary system is an essential part of the human body and includes the biliary tract and gallbladder. Its main function is to store and deliver the bile secreted by the liver to the duodenum. Similar to other systems of the human body, the biliary system is susceptible to carcinogenic changes. Cancers of the biliary tract are known as cholangiocarcinomas (CC) and are divided, based on the section of the tract, into intrahepatic (ICC) and extrahepatic CC (ECC) which further includes perihilar CC and distal CC.^1^ Although CC (ICC in specific) is generally less common than hepatocellular carcinoma (HCC), its incidence is on the rise and a strong association is observed with common risk factors such as chronic inflammations, bile stasis and cholelithiasis.^1^, ^2^ Currently, curative treatments are limited to surgical resection and challenged by the poor prognosis and high recurrence. Only 10% of patients maintain long‐term benefits of such surgical resections.^3^ Gallbladder carcinoma (GBC), on the other hand, is more common and ranks sixth among cancers of the digestive system. Late‐stage diagnosis, aggressive malignancy and poor prognosis are some of the troubling features that characterize this cancer which is known for its high incidence in China.^4^, ^5^ Similar to CC, surgical resection is the only curative approach for GBC and is usually challenged by a high recurrence rate (50%) that in some cases is encountered within weeks after resection.^6^

The challenges of high recurrence and poor prognosis created an essential need to develop an effective assessment system of surgical efficacy and post‐operative outcome. Such a system should enhance the prediction of prognosis and allow comparison among centers. For decades, assessment methods have been linked to single parameters such as mortality rate, morbidity, margin of resection or short‐term hospital readmission.^7^ Such factors provides useful insights to estimate the outcome of treatment. However, the single‐factor based assessment is usually influenced by the sample size and event rate and thus lacks the ability to comprehensively reflect the multidimensional influence of other potential factors. Therefore, similar assessments lack the ability to reflect differences between centers among which many conditions vary significantly.^8^, ^9^, ^10^

To address this limitation, a novel concept of assessment has been developed recently to provide a comprehensive multi‐dimensional prediction of short‐term postoperative prognosis, known as textbook outcome (TO). TO allows to base the assessment on several factors in an all‐or‐none approach, presenting an ideal concept of prognosis.^11^, ^12^, ^13^ So far, the definition of TO is still under development with no clear guideline to suggest included factors. Therefore, TO can vary based on the cancer type, surgery or even among centers. Nonetheless, few factors are generally agreed upon when setting TO criteria, such as short‐term mortality, hospital readmission, negative resection margin, postoperative complications and length of hospital stay after surgery.^11^, ^12^, ^13^, ^14^, ^15^

Studies addressing TO of the biliary system are relatively rare and the definition and rate of TO significantly vary among studies based on the location of cancer and surgical approaches.^7^, ^11^, ^12^ In this study (completed in accordance with the STROBE reporting checklist), we investigate the TO of biliary system cancers, provide a novel definition of TO, analyze TO influence on patients' survival and expand the concept of TO to address novel aspects of desired outcome that have never been acknowledged in previous relevant studies.

## 2 METHODS

### 2.1 TO definition

An “all or none” TO definition was adopted in this study and included the following criteria: (1) no re‐admission within 30 days after discharge; (2) no mortality within 30 days after discharge; (3) negative surgical margin (R0); (4) no severe postoperative complications (defined as Clavien–Dindo grade ≥III complications); (5) no prolonged hospital stay (≦50th percentile of patients, calculated separately for GBC and CC patients) (6) rapid decrease of total bilirubin (TB) to a level < 30 μmol/L (≦50th percentile of patients, calculated separately for GBC and CC patients); (7) rapid decrease of international normalized ratio (INR) to normal levels (0.85–1.5) (≦50th percentile of all patients); (8) rapid recovery or routine blood test (≦50th percentile of all patients): (a) rapid decrease of white blood cells (WBC) to a level ≦10×10^9^/L, (b) rapid increase of hemoglobin (HGB) to a level >100 g/L or an increase of 10 g/L without blood transfusion, (c) rapid recovery of platelet count (PLT) to normal levels (125 − 350 × 10^9^/L).

### 2.2 Patients' inclusion

Data of all patients who received treatment in China‐Japan friendship hospital between January, 2019 and March, 2023 were first reviewed and patients who fulfilled the following criteria were included in the study population: (1) confirmed clinical and pathological diagnosis of GBC or CC; (2) a surgical intervention with curative‐intention; (3) availability of all TO‐definition relevant data and survival status.

Data of age, sex, presence of chronic, or underlying diseases, preoperative biliary drainage, preoperative liver function (ALT, AST, TB, and DB), preoperative tumor markers (AFP, CEA, and CA199), preoperative routine blood test (WBC, HGB, and PLT), perioperative bleeding and blood transfusion, TNM staging, and neoadjuvant therapy application were then collected for included patients. In case of missing data, the total number of available cases was presented separately in relevant tables.

Fulfillment of TO relevant criteria was then evaluated and patients were divided into TO and non‐TO groups.

The study has been approved by the ethical committee of our hospital and carried in accordance with the Helsinki declaration. Due to the retrospective nature of the study and since no additional blood/tissue sample collection was required other than the routine follow‐up procedures, all patients were informed upon admission of the potential use of de‐identifying data for research and scientific purposes and the ethical committee approved the use of such data in the study.

### 2.3 Quality of life assessment

#### 2.3.1 Propensity score matching

Prior to carrying quality of life (QOL) assessment, PSM was carried between TO and non‐TO groups to limit the influence of irrelevant variables on the results. SPSS (v.25) was used to complete the matching with a 1:1 ratio and a 0.02 standard deviation range. Addressed variables included sex, age (≦65 years vs. >65 years), tumor type (GBC and CC), presence of chronic or underlying diseases, preoperative biliary drainage, perioperative bleeding (≦400 vs. >400 mL), blood transfusion, and the application of neoadjuvant therapy.

#### 2.3.2 QOL questionnaire

**FIGURE 1:**
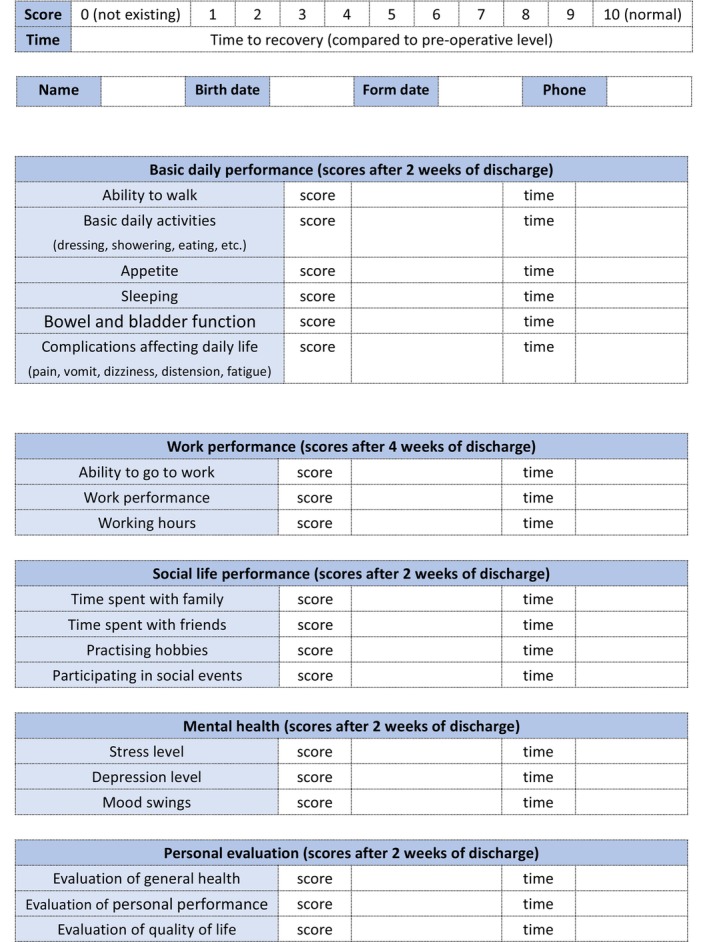
The customized questionnaire form for quality of life and all included items.

In order to complete QOL assessment, we developed a customized questionnaire to address the specific aspects of GBC and CC patients' short‐term recovery and post‐discharge life quality. Five main sections were included in the questionnaire (basic daily performance, work performance, social life performance, mental health and personal evaluation) with each section including several items. All sections focused on the recovery after 2 weeks of discharge except for the work performance assessment which focused on the recovery after 4 weeks. Each item of each section was graded on a scale from 0 to 10 with 10 points representing a full recovery to pre‐operative levels. When a grade lower than 10 was given, an exact time for full recovery was further provided. The final score of each section was calculated as the average of all items while the final score of the questionnaire was considered as the average of all sections' scores. The questionnaire form and all included items are shown in Figure 1.

### 2.4 Statistical analysis

SPSS (V.25, IBM) was used in this study to complete relevant statistical analyses. Continuous variables were presented as means ±standard deviations (SD) and compared by student *t*‐test while categorical variables were presented as numbers/percentages (%) and compared by chi‐squared test. Kaplan–Meier's method was used to compare survival curves while uni‐variate and multi‐variate logistic regression models were used to analyze the influence of relevant variables. PSM was carried as described earlier. Statistical significance of *p*‐values was considered when <0.05.

## 3 RESULTS

### 3.1 Study population and patients description

A total of 129 patients met the criteria and thus were included in this study. Of whom, 54 patients (41.86%) had GBC and 75 (58.14%) had CC. The TO rate was 25.58% (33 cases) for the general included population, 37.04% (20 cases) for GBC patients and 17.8% (13 cases) for CC patients. Several characteristics of patients differed significantly between TO and non‐TO groups such as sex, type of cancer, pre‐operative biliary drainage, preoperative liver function, preoperative tumor markers, perioperative bleeding and blood transfusion, and N stage. TO group tended to have higher rate of female patients (TO: 60.6%, non‐TO: 36.45%, *p* = 0.016), younger patients (≦60 years) (TO: 27.27%, non‐TO: 20.83%, *p* = 0.445), higher rate of GBC cases (TO: 60.6%, non‐TO: 35.41%, *p* = 0.011), lower rate of pre‐operative biliary drainage (TO: 9.09%, non‐TO: 28.12%, *p* = 0.026), higher rate of cases with normal preoperative liver function (TO: 84.37%, non‐TO: 36.17%, *p* < 0.001) and tumor markers (TO: 62.5%, non‐TO: 29.41%, *p* = 0.001), reduced perioperative bleeding (≦400 mL) (TO 90.9%:, non‐TO: 66.31%, *p* = 0.006) and thus reduced rate of blood transfusion (TO: 10%, non‐TO: 34.73%, *p* = 0.005), and higher rate of N0 stage cases (TO: 79.31%, non‐TO: 50.64%, *p* = 0.008). Detailed results are provided in Table 1.

**TABLE 1:** Patients characteristics in both TO and non‐TO groups.

*N* = 129	TO (*n* = 33)	Non‐TO (*n* = 96)	*p*‐value
Sex (M/F)	13/20	61/35	0.016
Age (≦60/>60) (year)	9/24	20/76	0.445
Type (GBC/CC)	20/13	34/62	0.011
Underlying diseases (y/n)	20/13	67/29	0.331
Preoperative biliary drainage (y/n)	3/30	27/69	0.026
Preoperative liver function (normal/abnormal) (*n* = 126)^a^	27/5 (*n* = 32)^a^	34/60 (*n* = 94)^a^	<0.001
Preoperative tumor markers (normal/abnormal) (*n* = 117)^a^	20/12 (*n* = 32)^a^	25/60 (*n* = 85)^a^	0.001
Preoperative routine blood test (normal/abnormal) (*n* = 122)^a^	23/10	66/23 (*n* = 89)^a^	0.622
perioperative bleeding (≦400/>400 mL) (*n* = 128)^a^	30/3	63/32 (*n* = 95)^a^	0.006
blood transfusion (y/n) (*n* = 128)^a^	3/30	33/62 (*n* = 95)^a^	0.005
T (IS+1/2 + 3 + 4) (*n* = 115)^a^	8/24 (*n* = 32)^a^	8/75 (*n* = 83)^a^	0.067
N (0/1 + 2) (*n* = 106)^a^	23/6 (*n* = 29)^a^	39/38 (*n* = 77)^a^	0.008
M (0/1) (*n* = 109)^a^	31/0 (*n* = 31)^a^	75/3 (*n* = 78)^a^	0.557
Neoadjuvant therapy (y/n) (*n* = 128)^a^	0/33	6/89 (*n* = 95)^a^	0.338

^a^ Indicates a change in total number due to missing data.

### 3.2 Survival analysis

**FIGURE 2:**
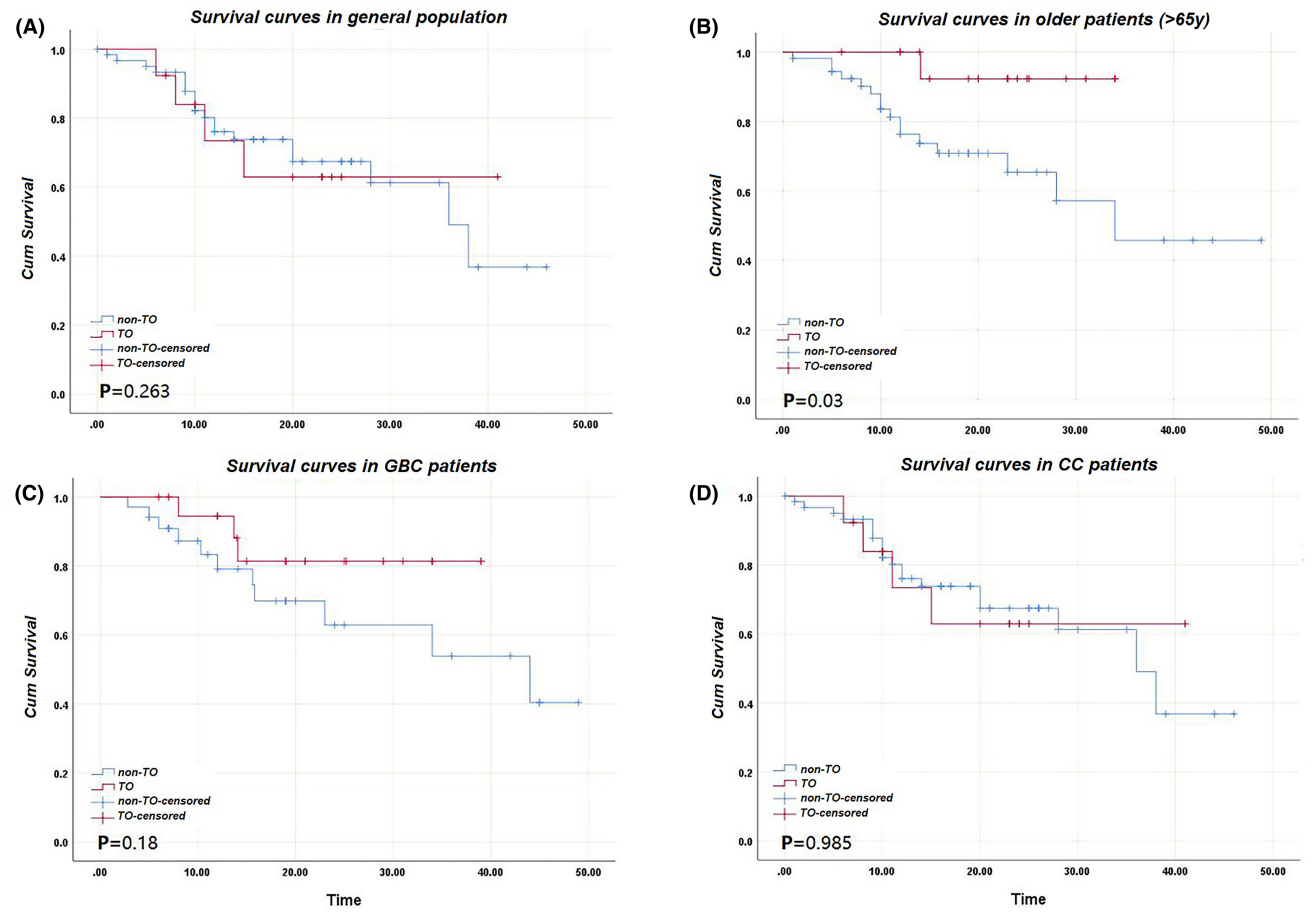
(A) Survival curves of TO and non‐TO groups in general study population; (B) survival curves of TO and non‐TO groups in patients older than 65 years; (C) survival curves of TO and non‐TO groups in GBC patients; (D) survival curves of TO and non‐TO groups in CC patients.

A better survival was observed in TO group when compared to non‐TO; however, no statistical significant was observed regarding the difference between the two groups. The 1‐year, 2‐year and 3‐year survival rates in TO group were 88.88%, 64.7% and 33.33%, respectively (compared to 78.66% (*p* = 0.242), 50.98% (*p* = 0.325) and 28.94% (*p* = 1) in non‐TO group). Survival curves of TO and non‐TO are shown in Figure 2A. A sub‐group analysis revealed a significant difference (*p* = 0.03) between TO and non‐TO in patients older than 65 years. The two curves are shown in Figure 2B. The one‐year and two‐year survival rates of TO versus Non‐TO groups were: 100% versus 78.57% (*p* = 0.108) and 87.5% versus 44% (*p* = 0.046). The limited number of patients prevented a significant calculation of three‐year survival. As for the tumor type based sub‐group analysis, enhanced survival (although statistically insignificant) was observed in TO group of GBC patients compared to non‐TO patients (Figure 2C), while no difference was observed between groups of CC patients (Figure 2D).

The influence of other variables on survival was also analyzed and only the influence of preoperative tumor markers level was significant in the multi‐variate analysis. Normal levels were associated with a decreased risk of death (HR (95% CI): 3.157 (1.145–8.709), *p* = 0.026). Detailed results are provided in Table 2.

**TABLE 2:** Uni‐variate and multi‐variate analyses of variables' influence on the risk of death.

*N* = 129	Uni‐variate analysis		Multi‐variate analysis (*n* = 96)^a^	
	HR (95% CI)	*p*‐value	HR (95% CI)	*p*‐value
Sex (M/F)	1.443 (0.723–2.878)	0.298		
Age (≦60/>60)	0.816 (0.368–1.812)	0.618		
Type (GBC/CC)	0.751 (0.384–1.466)	0.401		
Underlying diseases (N/Y)	0.593 (0.287–1.227)	0.159		
Preoperative biliary drainage (y/n)	0.745 (0.368–1.51)	0.414		
Preoperative liver function (normal/abnormal) (*n* = 126)^a^	0.691 (0.35–1.363)	0.286		
Preoperative Tumor markers (normal/abnormal) (*n* = 117)^a^	0.344 (0.150–0.791)	0.012	3.157 (1.145–8.709)	0.026
Preoperative routine blood test (normal/abnormal) (*n* = 122)^a^	1.048 (0.488–2.252)	0.904		
perioperative bleeding (≦400/>400 mL) (*n* = 128)^a^	0.601 (0.307–1.174)	0.136		
blood transfusion (y/n) (*n* = 128)^a^	0.557 (0.275–1.128)	0.104		
T (IS+1/2 + 3 + 4) (*n* = 115)^a^	0.518 (0.122–2.195)	0.372		
N (0/1 + 2) (*n* = 106)^a^	0.44 (0.21–0.922)	0.03	1.374 (0.621–3.041)	0.433
M (0/1) (*n* = 109)^a^	0.437 (0.103–1.85)	0.261		
Neoadjuvant therapy (y/n) (*n* = 128)^a^	0.495 (0.151–1.621)	0.245		

^a^ Indicates a change in total number due to missing data.

### 3.3 QOL assessment

Out of 129 patients included in the original study population, a total of 127 had complete data of the previously mentioned PSM addressed variables and thus were included in the pre‐PSM population (TO: 33 patients, non‐TO: 94 patients). The statistical comparison between TO and non‐TO groups in pre‐PSM population revealed significant differences regarding sex, tumor type, preoperative biliary drainage, perioperative bleeding and blood transfusion. After PSM, a total of 29 patients were included in each group and all addressed variables were statistically indifferent between the two groups. Details of pre‐PSM and post‐PSM comparisons are shown in Table 3.

**TABLE 3:** TO and non‐TO comparison in pre‐PSM and post‐PSM populations.

*N* = 127	Pre‐PSM analysis			Post‐PSM analysis		
	TO (*N* = 33)	Non‐TO (*N* = 94)	*p*‐value	TO (*N* = 29)	Non‐TO (*N* = 29)	*p*‐value
Sex (M/F)	13/20	60/34	0.015	13/16	14/15	0.792
Age (≦65/>65)	16/17	41/53	0.629	12/17	10/19	0.588
Type (GBC/CC)	20/13	32/62	0.008	17/12	21/8	0.269
Underlying diseases (n/y)	13/20	34/60	0.741	9/20	10/19	0.78
Preoperative biliary drainage (N/Y)	30/3	67/27	0.022	26/3	27/2	1
Perioperative bleedin (≦400 />400 mL)	30/3	62/32	0.006	26/3	26/3	1
Blood transfusion (N/Y)	30/3	62/32	0.006	26/3	25/4	1
Neoadjuvant therapy (N/Y)	33/0	88/6	0.338	29/0	29/0	1

The QOL comparison between TO and non‐TO groups in post‐PSM population revealed statistically significant differences in several aspects, in addition to the final score. Patients in TO group had better basic daily performance (*p* < 0.001), social life performance (*p* = 0.033) and enhanced personal evaluation (*p* < 0.001) than those in non‐TO group, which resulted in a higher QOL final score (TO: 9.6272 ± 0.63969, non‐TO: 8.7428 ± 9.1300, *p* < 0.001). Detailed scores of each section of QOL questionnaire are shown in Table 4.

**TABLE 4:** QOL comparison between TO and non‐TO groups in post‐PSM population.

	TO (*n* = 29)	Non‐T0 (*n* = 29)	*p*
Basic daily performance	9.3824 ± 0.61604	8.2445 ± 0.95629	<0.001
Social life performance	9.8103 ± 0.54139	9.2411 ± 1.24811	0.033
Mental health	9.7700 ± 0.82191	9.4476 ± 1.15942	0.227
Personal evaluation	9.5397 ± 0.89711	8.0783 ± 1.04457	<0.001
Final score	9.6272 ± 0.63969	8.7428 ± 0.91300	<0.001

## 4 DISCUSSION

Although the concept of TO has been addressed in several types of tumors, it is still considered relatively new and in need of further exploration and development. The rate of TO is usually influenced by the type of tumor, surgery and center of treatment. Currently, no relevant guideline is available to set a clear definition or inclusion criteria. As for tumors of the biliary system, only limited number of studies investigated TO of CC and even fewer studies focused on GBC. The definition of TO in biliary system tumors generally included aspects like short‐term mortality, hospital readmission, negative resection margin, postoperative complications and length of hospital stay after surgery.^11^, ^12^, ^13^, ^14^, ^15^ However, the clear differences in reported outcome among studies and centers led us to consider the need for a better detailed definition that would address aspects directly related to post‐surgical short‐term recovery. In this study, we provide for the first time a novel TO definition for biliary system tumors that build on the previous definitions, expand the scope of TO and consider the specific aspects of short‐term recovery of patients with CC and GBC. In addition to the previously mentioned criteria, our definition required a rapid decrease of TB, INR, WBC, HGB and PLT. Such indexes are closely monitored after surgeries of biliary system tumors and significantly reflect the course of short‐term recovery. Due to their significance, both INR and bilirubin levels have been included in The Model for End‐Stage Liver Disease (MELD) evaluation^16^ while routine blood tests have been linked to prognosis prediction and chances of complications after hepatic surgeries.^17^, ^18^ Acceptable ranges of indexes have been set in accordance to the ranges used in our clinical practice or based on clinical experiences. Considering the differences between CC and GBC (pre‐surgical jaundice levels, surgical complexity and need of longer hospital stay), GBC and CC patients' 50th percentiles were calculated separately for TB and hospital stay. The separate calculation also helped overcoming (to a certain extent) the influence of surgical heterogenicity on the analysis. Although different surgeries (mainly radical cholecystectomy, pancreatoduodenectomy and hepato‐pancreatoduodenectomy) were used in different cases, the TO definition criteria of no re‐admission, no mortality, R0, and no severe postoperative complications (grade ≥III) are appropriate regardless to the surgical approach. The rapid recovery of routine blood tests and coagulation function may be influenced by the perioperative bleeding and extent of damage to the liver; however, such factors are compensated for during surgery by blood transfusion or after surgery through liver protective medication. In addition, perioperative bleeding has been included in our analysis to limit its influence on the results. Therefore, only TB levels and hospital stay in TO definition are directly influenced by surgery (and thus type of tumor).

Although our definition may appear complex to some reviewers, we believe it is very easy and feasible in clinical practice, as the original criteria basically include data collected in any follow‐up protocol, while the novel criteria only require collecting blood samples after surgery. Both procedures (blood monitoring and follow‐up) are necessary steps of any treatment strategy regardless to the center or region.

Our study reported TO rate of 25.58% for all patients, 37.04% for GBC patients and 17.8% for CC patients which further reflect the obvious differences between cancer types. Such differences fall in line with results of previous studies as GBC TO rates are usually higher (as high as 41.3%^12^) than CC (as low as 11.2% for ICC^7^). The comparison of patients characteristics between TO and non‐TO group similarly revealed a higher rate of GBC patients in TO group (60.6%) compared to non‐TO (35.41%) (*p* = 0.011). TO group also had fewer cases of preoperative biliary drainage (*p* = 0.026), more cases with normal preoperative liver function (*p* < 0.001) and tumor markers (*p* = 0.001), limited perioperative bleeding (*p* < 0.001) and blood transfusion (*p* = 0.005), and lower rate of lymph node metastasis (*p* = 0.008). The differences between the two groups reveal a better preoperative condition of GBC patients and explain the difference in TO rates.

In addition to differences in patients' characteristics, our survival analysis revealed better one‐year, two‐year, and three‐year survival rates (88.88%, 64.7%, and 33.33%, respectively, compared to 78.66%, 50.98%, and 28.94% in non‐TO group); however, the difference was statistically insignificant, probably due to the influence of sample size. Considering the potential differences in survival, we carried a sub‐group analysis based on tumor type (GBC and CC separately) and patients' age ((≦65/>65)). A significant difference was observed in patients older than 65 years (*p* = 0.03). The one‐year and two‐year survival rates of TO versus non‐TO groups were: 100% versus 78.57% (*p* = 0.108) and 87.5% versus 44% (*p* = 0.046), respectively, which may indicate that our novel definition of TO is a better fit and more sensitive to reflect the survival of older patients. No significant differences were observed in GBC and CC groups separately. Due to the limited number of patients, we could not carry a further comparison in older GBC and CC patients, yet we believe future investigations with larger number of patients can address this point and potentially reveal significant differences.

As mentioned earlier, the main aim of this study was to expand the concept of TO in patients with biliary system tumors, enhance its ability to reflect the post‐surgical recovery and address novel, yet essential, aspects that have not been considered by relevant investigations before. Therefore, in addition to the novel definition and its influence on survival, we also focused in this study on exploring the QOL of patients and its relation with TO. We believe in our clinical practice that patients' recovery is an integrated concept that should never be limited to surgical or physical factors, a full recovery should always include the improvement of patients' psychological well‐being and functioning in daily life. Thus, to our knowledge, we are the first to investigate the connection between TO and QOL of patients. Many QOL questionnaires are already available for cancer patients and cover essential aspects of patients ability and functioning, such as European Organization for Research and Treatment of Cancer QLQ‐C30 (EORTC‐QLQ‐C30).^19^ Considering the specificity of biliary system surgeries, and building on previously mentioned questionnaires, we developed a new customized questionnaire to address the specific aspects of GBC and CC patients' short‐term recovery and post‐discharge life quality. The questionnaire focused on five aspects of patients' post‐discharge performance: basic daily performance, work performance, social life performance, mental health and personal evaluation. However, we decided not to include the results of work performance as the majority of included patients are above the age of retirement. The timeline was set as 2 weeks after discharge to reflect the short‐term recovery. In addition, for the majority of patients, post‐surgery adjuvant treatment is usually initiated after 3–4 weeks of surgery. Thus, we wanted to eliminate the potential influence of adjuvant treatment on the results. PSM with a 1:1 ratio and a 0.02 standard deviation range was carried to eliminate the influence of sex, age (≦65 years vs. >65 years), tumor type (GBC, CC), presence of chronic or underlying diseases, preoperative biliary drainage, perioperative bleeding (≦400 vs. >400 mL), blood transfusion, and the application of neoadjuvant therapy on results. We chose the ratio of 1:1 and not 1:2 or 1:3 as we wanted to minimize the bias at the cost of losing cases. However, future studies with larger sample sizes can further explore the topic with different ratios based on each study's design, sample size and preferences. Our findings revealed that the novel TO definition is indeed able to reflect the enhancement of patients' QOL and significantly better basic daily performance (*p* < 0.001), social life performance (*p* = 0.033), enhanced personal evaluation (*p* < 0.001) and QOL final score (*p* < 0.001) were observed in TO group when compared to non‐TO. Higher scores of social life performance and mental health evaluation were also observed in TO group; however, the differences were statistically insignificant. Such insignificant difference is actually expected considering the close involvement of family in the post‐discharge daily life of patients (which explain the insignificant difference in social life performance) and the fact that most patients of older ages are usually unaware of many details of their conditions and treatment outcome as family members prefer to handle such details to reduce patients mental and psychological burden.

During the completion of this study, certain challenges and limitations have been faced. For example, our data are a presentation of a single‐center outcome. Thus, future studies can further adopt multi‐center designs to further compare the efficacy of new TO definition in comparing different centers' quality of treatment. Other challenges, like the retrospective nature of the analysis and the influence of patients number on the study design prevented us from carrying further analysis in some sections. Another point to mention is the suggestion of excluding Tis stage from the study. Only one case in our study was categorized post‐operatively as Tis. Nonetheless, the patient received a standard pancreatoduodenectomy based on the preoperative evaluation. Thus, we included the case in our study as we believe it is appropriate and fits the scope of the topic discussed. Similarly, the study included three cases of metastasis regardless to the curative surgical intention stated earlier. Such cases of metastasis are diagnosed during surgery as previously undetected metastatic lesions to the abdominal wall or neighboring organs are discovered.

To conclude, our findings proved to be valuable and significant. Achieving the aim of the study, we provided for the first time a novel TO definition able to better address the specific nature of recovery after surgical resection of GBC and CC. We investigated the influence of TO on survival of patients and observed a greater influence in older patients. We also expanded the scope of TO concept by addressing, for the first time, the essential connection between TO and QOL of patients, providing a novel comprehensive approach that we believe is extremely important to direct future development of TO concept in biliary system tumors.

## AUTHOR CONTRIBUTIONS

**Manar Mikhail Atyah:** Conceptualization (lead); data curation (lead); formal analysis (lead); investigation (equal); methodology (equal); project administration (equal); software (equal); validation (equal); visualization (equal); writing – original draft (lead); writing – review and editing (lead). **Li Xu:** Conceptualization (equal); formal analysis (equal); investigation (equal); methodology (equal); project administration (equal); resources (equal); supervision (equal); validation (equal); visualization (equal); writing – original draft (supporting); writing – review and editing (equal). **Zhiying Yang:** Conceptualization (supporting); funding acquisition (lead); investigation (supporting); methodology (supporting); project administration (equal); resources (lead); supervision (equal); writing – review and editing (supporting).

## FUNDING INFORMATION

This work was supported by the National Key Clinical Specialty Construction Project General Surgery (Hepatobiliary Surgery) (2021‐QLT‐004).

## CONFLICT OF INTEREST STATEMENT

All authors declare no conflict of interest.

## ETHICS STATEMENT

The authors are accountable for all aspects of the work in ensuring that questions related to the accuracy or integrity of any part of the work are appropriately investigated and resolved.

## Data Availability

The data that support the findings of this study are available from the corresponding author upon reasonable request.
